# Teaching Medical Students How to Use Interpreters: A Three Year Experience

**DOI:** 10.3885/meo.2009.Res00309

**Published:** 2009-09-02

**Authors:** Mimi McEvoy, Maria Teresa Santos, Maria Marzan, Eric H. Green, Felise B. Milan

**Affiliations:** * Department of Pediatrics; † Department of Family Medicine; ‡ Department of Medicine, Albert Einstein College of Medicine Bronx, New York 10461

**Keywords:** Interpreters, limited English proficiency, cultural competency, medical education

## Abstract

Disparities in health exist among ethnic/racial groups, especially among members with limited English proficiency (LEP). The session described in this paper aimed to teach medical students the skills needed to communicate with patients with LEP. *Description* – We created a required session titled “*Cross-Cultural Communication-Using an Interpreter*” for third-year medical students with learning objectives and teaching strategies. The session plans evolved over three years. *Program Evaluation* – Students’ perceived efficacy using retrospective pre/post test analysis (n = 110, 86% response rate) administered 7 weeks post-session revealed that 77.3% of students felt “more prepared to communicate with a patient with LEP”, 77.3% to “give proper instructions to an untrained interpreter” and 76.4% to “access a hospital language line”. *Conclusion* – Our curricular intervention was effective in increasing students’ perceived efficacy in communicating with a patient with LEP, using untrained interpreters and accessing a hospital language line. Skills practice and discussion of using interpreters should be a part of medical education.

Disparities in health exist among ethnic/racial groups, especially among members with limited English proficiency (LEP).^1^ Studies show that patients with LEP are less likely to receive adequate referrals for health prevention, including health educational counseling,^2^ and may undergo more diagnostic testing.^3^ They are also at higher risk for non-adherence to treatment recommendations,^4^,^5^ receiving inadequate information for medical consent,^6^ having greater dissatisfaction with care,^7^ and for medical errors.^8^ About 45 million people in the US speak a language other than English at home, and over 30 million people in this country were born outside the US.^9^ Providers need to acquire cross cultural communication skills and provide interpreter services to meet the challenges of communicating with a linguistically diverse population.

In response to a lack of comprehensive standards to address these issues, the Office of Minority Health (OMH) published the National Standards for Culturally and Linguistically Appropriate Services (CLAS) in Health Care in 2001.^10^ The 14 CLAS standards, directed primarily at health care organizations, state that health care providers need to offer patients in their preferred language both verbal and written notices of their right to receive language assistance and services, including bilingual staff and interpreter services. These standards have fueled the impetus for medical schools to teach students the skills to use interpreters in order to effectively communicate with patients with LEP.

Many schools have combined curricular efforts to teach students how to use interpreters with cultural competency and medical Spanish programs.^11^,^12^ It is still unclear, however, where in the curriculum this content should be placed. Many medical schools relegate these topics to the pre-clinical years, though it is during the clinical clerkships when students will need to implement these skills. We felt that placing our session into the early sessions of our third year Patients, Doctors and Communities (PDC) course would be ideal for two reasons: A large part of our patient population in the Bronx have LEP, and a major responsibility of third year students is to interview patients. In this paper, we describe a session, “Cross Cultural Communication-Using an Interpreter”, that we have honed over the past 3 years to equip students with knowledge and skills to use interpreters.

## Description

At the Albert Einstein College of Medicine, we have a 16-session longitudinal course called Patients, Doctors and Communities (PDC) that runs concurrently with the third year clerkships and includes 4 introductory sessions in the spring of the second year before students start their clerkships. We created a session titled *Cross-Cultural Communication-Using an Interpreter* as part of the PDC curriculum. The overall goals/objectives of this session are to help students:

1. Understand the principles of communicating with culturally diverse patients with LEP
2. Practice the skills of using an interpreter.

Our students encounter an ethnically and linguistically diverse population in the Bronx, New York. Thus, as course leaders, we recognized that we had both a responsibility and an opportunity to teach our medical students how to communicate with a patient and/or family with LEP. In planning for this session 3 years ago, a committee of experts was convened. The committee included a Family Medicine clinician, two general internists, a nurse practitioner and a health educator, all of whom had extensive experience working with patients with LEP. We have also had the same curriculum committee for this session for the past 3 years which has provided continuity throughout the planning process.

At this point, we have 3 years of experience in conducting this required session (2005–2007). In the first two years (2005 and 2006), we included this session as one of 4 introductory sessions within the PDC course that occurs approximately 6 weeks before students begin their clerkships. In the third year (2007), we moved this session from the 4 introductory sessions into the early part of the actual course during the first week of students’ clerkships. In order to describe the evolution of the course, we will present each of the 3 years separately.

**Year 1** - In the first year, we had 3 overall objectives:

1. Understand and identify the challenges and issues in communicating with patients from diverse backgrounds
2. Identify the appropriate techniques when utilizing an interpreter
3. Understand the importance of interpreters to effectively communicate with patients with LEP.

We divided the 2-hour session into 2 equal parts. One hour focused on understanding various health beliefs of patients and families, how those beliefs impact health care delivery, and the complexities of communicating with patients about health beliefs when language is a factor. We used a trigger video, “Mr. Kochi's Story”,^13^ about an Islamic patient with cancer who speaks only Farsi. The story illustrates the cascade of communication problems that ensued between the patient, his family and the healthcare providers because of language barriers. In the second hour, we showed a short video outlining the “dos and don'ts” of using untrained interpreters.^14^ Then, we asked the students to apply this information by viewing and critiquing 2 other short video vignettes showing interpreter use: a mother from Puerto Rico who has a child with asthma and a elderly man from Haiti with vague abdominal pain and sore joints, which he refers to as “gaz”.

**Year 2** - In the second year, we added 2 objectives and teaching strategies, specifically including skills practice:

1. Demonstrate the ability to elicit a patient's perspective and health beliefs surrounding a problem or illness
2. Describe and demonstrate the ability to use an untrained interpreter effectively.

We made these changes because we understood from the literature that experiential learning (i.e., skills practice) is effective. In the session's first hour, we used the same video as in year 1 to discuss principles of interpreter use. We also viewed and briefly discussed the video vignettes illustrating the appropriate use of both professional and untrained interpreters by comparing and contrasting the technical aspects of the interview in each vignette. In the second hour, we provided an opportunity for students to practice these skills. We recruited 11 Spanish-speaking patients from our school's Medical Spanish Program and 11 medical Spanish teachers from this same program to act as untrained interpreters. None of these teachers had been trained in medical interpretation. Each interpreter-patient pair worked with a group of 8 students and their faculty facilitator for 1 hour. Students were instructed to practice interviewing the patient using the untrained interpreter by focusing on the history of present illness and social history within the context of the patient's cultural and spiritual beliefs. For example, students would ask the patient to explain his/her understanding of any current medical problem and to talk about a treatment plan. If the patient was comfortable disclosing his/her use of complementary and alternative therapies, students were encouraged to engage the patient in a discussion. At different points during the interview, the facilitator and student interviewer paused to discuss the interview's progress. The student also received feedback on skills from the observing students, facilitator and untrained interpreter. The cycle was repeated with as many students getting a chance to interview as time allowed. Table 1 describes the process for giving multiple students an opportunity to interview.

**Table 1.** Guidelines for facilitating small group skills practice in working with an interpreter and Spanish-speaking patient

Facilitate a brief discussion of past experiences using an interpreter in the medical setting. If students have none to share, you may want to share your own experience(s). Introduce skills practice participants: Spanish speaking (real) patient and Spanish language interpreter (AECOM Medical Spanish instructor or Internal Medicine resident). The patients have been instructed to pretend that this is an office visit but to be themselves and discuss an actual medical problem that they have had or have currently. Explain the goals of the skills practice session and set the agenda. Reinforce the idea that “we're all in this together” and “we can learn from each other” and that mistakes are learning opportunities. Set the ground rules. You may want to acknowledge that interviewing in front of a group is hard; the interviewer is in a vulnerable position. Reinforce that mutual respect and constructive comments are necessary for a comfortable learning environment. Remind them of the “feedback” sandwich (starting and ending with positive feedback to “soften” the impact of more negative statements) and the need to balance positive and constructive comments. Strongly suggest that the group is responsible for providing feedback for each other. You will be facilitating this process. **(Note 6 and 7 can be done in either order)** Ask for (or choose) a student to volunteer to begin the interview (who goes first?) Ask the group or first interviewer to arrange the chairs for skills practice exercise. This should be done with attention to relative positioning of patient, interviewer and interpreter. Round Robin Role Play - The role play begins with a student interviewing the patient. **Time outs** can be called by either faculty or interviewer for the following reasons……time to give someone else a turn, interviewer is stuck or opportunity for a great learning point, etc. **Feedback**…When role play is paused (time out called) let the interviewer speak first, allow the group to share their ideas/suggestions then share your own. You can ask the group to work together to problem solve if there is a “stuck moment”. Normalize and universalize the challenges of the bilingual interview. The interviewer may want an opportunity to correct mistakes or you can move on to next interviewer. You can ask for feedback from the interpreter or from the patient through the interpreter. **Rotate** the role of the interviewer until many of the students have had a chance to interview the patient using the interpreter. It usually works best for the next interviewer to pick up where the last one has left off. (“Dr. X had to go; I'll be taking his/her place”). If the group wishes to do this differently, feel free to experiment. Once all have had a turn or time is getting short, conclude with a review/summary of the learning points experienced by the group.

**Year 3** - In the third year, we changed the session based on faculty feedback during post-session discussions from the previous year. We modified the session's timing to occur during the first week of the third year clerkship. The change was intended to provide students with an opportunity to apply the practical skills sooner since we anticipated that most students would encounter a patient with LEP at the clinical sites within their first week. The other substantive change was the addition of instruction on using telephone interpreter services, a service now used in all affiliate hospitals. We thought that it was practical and would be more effective to prepare students with the skills that they would most likely be using during their clerkship experience. With patient permission, we videotaped an interview of a patient and physician encounter that required the use of a telephone interpreter at a clinical affiliate site. Students watched this video during class and then discussed the interview, including the similarities and differences among using an untrained, professional and telephone interpreter. We also supplied students with a small card listing the phone access codes to the telephone interpreter services for all affiliate hospitals. The discussion of “Mr. Kochi's Story” was moved into the following session in the PDC course on health beliefs. We instructed students to watch this video from the school's intranet to prepare for that session.

The session's evolution during our 3 year experience of changing learning objectives and teaching strategies is summarized in Table 2.

**Table 2.** Evolution of the Curriculum-“*Cross Cultural Communication – Using an Interpreter*” Session

	Learning Objectives	Teaching Strategies	Outcome Measures
**Year 1**	Understand and identify the challenges and issues (i.e. gender, religion, language) in communicating with patients from diverse backgrounds	View and discuss the video, “World's Apart: Mr. Kochi's Story”	Students’ perception of session effectiveness
	Identify the appropriate techniques when utilizing an interpreter during a patient interview	View and discuss a video outlining the principles of using an untrained interpreter	
	Understand the importance of interpreters to effectively communicate with patients with LEP	View and discuss a comparison of 2 video vignettes using interpreters for patients with LEP	
**Year 2**	Understand and identify the challenges and issues (i.e. gender, religion, language) in communicating with patients from diverse backgrounds	View and discuss the video, “World's Apart: Mr. Kochi's Story”	Students’ perception of session effectiveness
	Identify the appropriate techniques when utilizing an interpreter during a patient interview	View and discuss a video outlining the principles of using an untrained interpreter	
	Demonstrate the ability to elicit a patient's perspective and health beliefs of a problem/illness Demonstrate the ability to use an interpreter effectively	Practice the skills of eliciting a history of present illness within the context of culture from a Spanish speaking patient using an untrained interpreter	
**Year 3**	Identify and describe the appropriate techniques when utilizing an interpreter during a patient interview	View and discuss a video outlining the principles of using an untrained interpreter	Students’ perceived efficacy using retrospective pre/post test analysis
	Demonstrate the ability to elicit a patient's perspective and health beliefs of a problem/illness Describe and demonstrate the ability to use an interpreter effectively	Practice the skills of eliciting a history of present illness within the context of culture from a Spanish speaking patient using an untrained interpreter	
	Learn the skills of accessing a telephone interpreter line	View and discuss an internally made videotape of a doctor-patient encounter using a telephone interpreter with an actual patient with LEP	
	Link principles and skills learned in this session to the subsequent session on “Health Beliefs”	Post-assign the video of “Mr. Kochi's Story” to be viewed subsequent to the session on “Health Beliefs”	

## Program Evaluation

In 2007 we administered a 3-item retrospective pre/post survey 7 weeks after the session to 128 students to measure the session's effect on their perceived efficacy to use interpreters for communicating with patients with LEP. The survey used a 5-point Likert scale (1 = strongly disagree; 2 = disagree; 3 = uncertain; 4 = agree; 5 = strongly agree) for rating the items. The prompt “I feel more prepared now than I did before the session (name of session) on (date) to:” was used to elicit responses to these items:

1. Communicate with a patient who has limited English skills
2. Give instructions to an untrained interpreter
3. Access a hospital language line.

**Figure 1.**
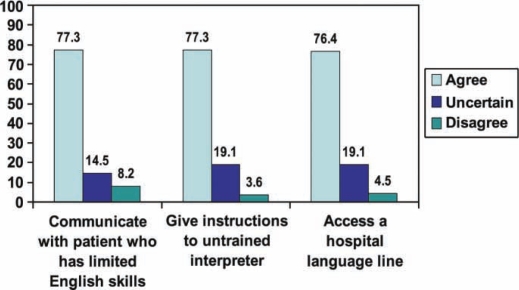
Percentage of third-year students who agreed, uncertain, or disagreed in a retrospective pre/post self-assessment ratings of skills 7 weeks after session.

Administering the survey 7 weeks after the session allowed the majority of the class to have interviewed a patient with LEP by that time. We received 110 surveys (response rate = 86%). For analysis the scores for strongly agree and agree were combined, with strongly disagree and disagree treated the same way. Figure 1 shows that 77.3% of students agreed or strongly agreed that they “felt more prepared now than before the session to communicate with a patient who has LEP”; 14.5% were uncertain. The same percentage of students (77.3%) either agreed or strongly agreed that they “felt more prepared to give instructions to an untrained interpreter”, with 19.1% being uncertain. 76.4% of students agreed or strongly agreed that they “felt more prepared to access a hospital language line”; 19.1% indicated uncertainty. Only a small percentage of students (8.2%) disagreed or strongly disagreed with any of the 3 items.

Students rated the session highly on course evaluations in all 3 years. The positive results on the retrospective pre/post surveys in 2007 agreed with the students’ positive ratings of the session. The session received a mean overall rating of 4.1 (out of 5) in 2005 (n = 159), 4.3 in 2006 (n = 154), and 4.4 in 2007 (n = 155), based on a scale where 1 = strongly disagree and 5 = strongly agree in terms of accomplishing session objectives. Anecdotal comments revealed that students appreciated the opportunity to practice interviewing patients with LEP, e.g, “I still use the guidelines [for using an interpreter] in interviewing patients today.”

## Discussion and Conclusions

The large majority of students agreed retrospectively that they felt better prepared to communicate with a patient with LEP, give instructions to an untrained interpreter and access a hospital language line. While others have developed methodologies to demonstrate programmatic effectiveness using standardized patients,^15^,^16^ pre/post tests,^17^ and bi-lingual medical students,^18^ we measured students’ perceived efficacy 7 weeks after the teaching session when they would have had an opportunity to apply the skills with actual patients with LEP in their clerkships. Retrospective pre/post analysis has been shown to be a reliable method of measuring skills as demonstrated by Skeff and colleagues in 1992.^19^,^20^ Given that students perceived themselves to be more prepared in all 3 measures (communicating with a patient with LEP, giving instructions to an untrained interpreter, and accessing a hospital language line) after this training session, it is a fair assumption that the intervention was effective in having students apply the skills successfully in the clinical setting. Course evaluations administered in all 3 years confirmed that students perceived the session to be helpful. The small percentage of students who were uncertain of the effects of the session on all these items were not further questioned about the reasons. Perhaps they had not yet encountered a patient with LEP or were actually uncertain about the session's usefulness.

The students who commented on using the skills in their clerkships reinforced for us that the placement of this session in the beginning of the third year is appropriate. A 2004 national survey of residents regarding their use of professional and non-professional interpreters showed that 35% received no instruction or very little instruction from their institutions on how to deliver care through interpreters. Yet 84% used untrained interpreters (family and friends), and 22% admitted using children to interpret.^21^ Hence, providing skills on how to use interpreters at the beginning of the clerkships may be the only opportunity trainees will have to get this training for situations that they are likely to encounter.

When to best teach students or residents about interpreter use is one issue, but how and what to teach is perhaps even more important. Because faculty planners were the same for all three years, we had the benefit of using experience and consistency to make curricular improvements and use student evaluations and faculty input each year. By the third year of teaching this topic, we concluded that both skills practice and discussion of health beliefs were important and deserved separate sessions. This separation allowed us to spend more time on the skills practice of using interpreters and to add the element of accessing a telephone interpreter. Many hospitals have language lines today, so familiarizing students with this service has relevance to their present and future practice.

Since physicians still have misconceptions about the effectiveness and the time constraints of using interpreters, practice sessions on interpreter use are not only useful for skills building, but may also dispel misconceptions about the logistics and time requirements. Rosenberg et al.^22^ studied physicians’ perceptions of using interpreters, finding that physicians perceived communication tasks to be more difficult when using an interpreter than when one was not needed. Tocher and Larson^23^ found similar results in a study of physicians who perceived that they spent more time with non-English-speaking patients than with English-speaking patients. Both studies, however, found no difference in the office visit time between the 2 patient types.

Methods for teaching medical students how to use an interpreter are still evolving, and studies on this topic are sparse. Teaching medical students these skills is important. Teaching strategies need continual development. Our next steps are therefore to investigate the session's behavioral outcomes for students who work with patients with LEP to determine if students use both trained and untrained interpreters properly. We plan to conduct focus groups to gain information on students’ clerkship experiences and devise methods to measure their actual performance in communicating with patients with LEP. We also need to investigate the prevalence of interpreters at the clinical sites. We do not know what role models exist at the clinical sites where students are really learning to communicate with patients with LEP and whether proper or improper use of trained or untrained interpreters is being reinforced. Hence, we need to find out if our students are actually able to apply the knowledge and skills with which we are equipping them. This latter point is the major barrier that exists. Until the curriculum and reality of medical practice mesh, we might still be working against the tide.

Our curricular experience, however, has shown us that the session we described is effective in increasing students’ perceived efficacy for using the skills to communicate with a patient with LEP, give instructions to an untrained interpreter and access a telephone interpreter. Since it is unlikely that students will receive the opportunity to formally learn these skills during residency, it is practical to equip them with the skills early in their medical school clinical training and, therefore, help to change the culture of proper interpreter use.
